# Impact of Microbial Strain on the Nitrogen Fixation of Soybean Varieties of Different Maturity Under Cool Climate Conditions of Northern Europe

**DOI:** 10.3390/plants14193097

**Published:** 2025-10-08

**Authors:** Raminta Skipitytė, Rūta Barisevičiūtė, Monika Toleikienė

**Affiliations:** 1 Institute of Agriculture, Lithuanian Research Centre for Agriculture and Forestry, Instituto av. 1, LT-58344 Akademija, Lithuania; monika.toleikiene@lammc.lt; 2 Center for Physical Sciences and Technology, Savanorių av. 231, LT-02300 Vilnius, Lithuania; ruta.bariseviciute@ftmc.lt

**Keywords:** *Glycine max* L., *Bradyrhizobium japonicum*, inoculation, stable nitrogen isotopes, organic farming

## Abstract

Soybean inoculation with nitrogen (N) fixing bacteria can be highly promising for enhancing biological nitrogen fixation (BNF) and improving crop productivity. It helps to reduce dependency on chemical fertilizers, promotes sustainable agricultural practices, and minimizes environmental impacts. Therefore, understanding the specific aspects and conditions is essential for establishing the BNF process in particular environments. In this study, we investigated whether soybean inoculation is an effective strategy in cool-climate regions beyond their typical northern distribution, and which soybean varieties and microbial strains are the most effective for optimizing soybean productivity and performance in relatively cool environments. To address these questions, a natural abundance nitrogen stable isotope ratio analysis was conducted on two soybean varieties of different maturity groups, which were inoculated with three *Bradyrhizobium japonicum* strains, along with organic fertilizer and new promising endophyte treatments. This approach aimed to determine the differences in biological and chemical parameters of soybean, as well as the origin of N sources, its uptake, and the isotopic distribution within the plants. It was demonstrated that inoculation with *Bradyrhizobium japonicum* was more effective than fertilization, as the strains had a significant effect on nitrogen derived from the atmosphere (Ndfa), produced stable nitrogen isotope ratios close to 0‰, and substantially increased nitrogen content, particularly in beans. Soybean varieties Laulema and Merlin, representing different maturity groups, exhibited distinct nitrogen uptake patterns. *Bradyrhizobium japonicum* strain *AGF78* consistently produced the greatest effect on biological parameters in both varieties, particularly in seed yield and grain weight, with the later-maturing Merlin achieving the highest average yield of 3066.89 kg ha^−1^. Notably, the Merlin inoculated with *AGF78* resulted in the highest nitrogen fixation in beans, with 66.8%NDFA and 134.0 kg/ha of fixed nitrogen. Similarly, Laulema inoculated with *AGF78* resulted in 88.2%NDFA and 123.2 kg/ha of fixed nitrogen. Inoculation with selected bacterial strains significantly increased protein content from 30% to 41%, with the effects being both strain- and variety-specific. Our study showed that establishing effective soybean–microbe interactions by choosing soybean variety and microbial strain is crucial for optimizing agricultural practices and improving crop performance, especially in sustainable and environmentally conscious farming systems under cool climatic conditions of Europe.

## 1. Introduction

The symbiotic nitrogen-fixing activity of legumes plays a crucial role in organic farming worldwide, enhancing soil fertility and reducing the need for chemical fertilizers [1]. High sensitivity of legume plants such as field pea and fababean to biotic and abiotic stresses poses a threat to the quality of legume beans, leading to lower yields and nutritional content [2], which may discourage farmers from choosing traditional legumes for cultivation. Therefore, implementing adaptation strategies for more diversified legume species is necessary to mitigate the climatic impact on grain legumes [3]. Among all legumes, grain legumes such as soybeans (*Glycine max* (L.) Merr.) are particularly important, as they can accumulate the highest protein content in the beans among other crops [4]. Ray et al. [5] showed that soybeans are typically cultivated in Europe with a latitude of up to 49°N. In less favorable regions for soybean cultivation, like Lithuania, situated beyond this northern limit, at 55°24′ N, there is still a significant potential for atmospheric nitrogen accumulation by soybean, which could further benefit sustainable agriculture [6]. Increasing crop diversity by including perennial legumes is an agrotechnical practice that strongly affects the soil environment [7]. However, due to the absence of indigenous soybean-nodulating bacteria in the soil of many regions, inoculation is essential for maximizing the biological nitrogen fixation potential of soybean. Therefore, studying the interactions between symbiotic microorganisms and soybean varieties is essential to focus on in order to improve nitrogen fixation efficiency and achieve higher yields in non-typical soybean growing regions, especially under cool temperate climatic conditions in the EU [8].

Key research on inoculation highlights the importance of utilizing effective bacterial strains to enhance productivity in regions with specific climatic conditions. Some notable studies have shown that *Bradyrhizobium japonicum* strains play a critical role in enhancing soybean yields. For instance, a study in Northeast Germany [9] found that inoculating soybeans with specific *Bradyrhizobium japonicum* strains significantly improved shoot dry weight and nitrogen uptake under field conditions, though the effectiveness varied depending on the cultivar. Similarly, research in Northern Ghana demonstrated that inoculants significantly increased both nodule formation and grain yield in soybean, making inoculation a viable strategy for enhancing productivity in challenging environments [10]. A study in Scotland showed [11] that inoculation doubled the grain yield to 1 t ha^−1^ compared to the non-inoculated crop, and inoculated soybeans obtained most of their nitrogen through nitrogen fixation. Toleikienė et al. [12] analyzed pre-crop effects of soybean in comparison to traditional grain and forage legumes. Comparing grain legumes, only efficiently nodulated soybeans produced a positive +20.6 kg ha^−1^ net N balance and increased the yield of subsequent spring wheat by 920 kg ha^−1^. These studies emphasize the potential of microbial inoculation to improve nitrogen fixation and plant growth, especially when choosing appropriate bacterial strains and considering local environmental factors. However, these types of studies in higher latitudes are still sparse [13].

Research on soybean genotypes has reported ambiguous results regarding their influence on nitrogen fixation efficiency and uptake [6,12,13]. Location, local climatic conditions, species adaptability, and maturity group appear to be the main factors influencing genotype responses to inoculation with different microbial strains and the resulting performance. Therefore, studies on adapted varieties highlight the potential to identify the most promising soybean genotype–microbial strain combinations that could be introduced into northern regions to ensure high protein yields while meeting most nitrogen requirements through biological nitrogen fixation (BNF). However, soybean cultivation remains relatively uncommon in Europe, especially in regions with colder climates. In Central to Northern Europe, the development of early-maturing soybean varieties adapted to cool growing conditions is still under investigation. In Central Europe, soybean cultivation remains insufficiently explored, and breeding efforts for early-maturing, cool-adapted varieties have only recently begun [14]. However, studies show that the soybean was successfully cultivated in even northern countries [13]. It is already confirmed that effective inoculation with *Bradyrhizobium japonicum* strains can significantly increase grain yield, protein content, and protein yield of various genotypes [14]. In Lithuania, soybean studies began several years ago. Soybean development and productivity were investigated by Toleikienė, Brophy, Arlauskienė, Rasmussen, Gecaitė, and Kadžiulienė [12]. This study suggests that regular soybean development could be maintained in organically managed locations above the present northern distributional region, but its development, productivity, and production quality significantly depend on management practices.

Since efforts need to continue to improve the nitrogen use efficiency (NUE) and to lower the undesirable environmental impact arising from N loss processes in agriculture, it is our proposition to investigate sources of BNF in legume plants and provide improved environmental outcomes compared to cropping systems reliant on fertilizer N. We propose that BNF should play a larger role in supporting the future projected growth in crop production. Thus, the aim of this study was to assess two varieties of soybean inoculated with different commercially available inoculants and identify the most effective pairs in terms of biological nitrogen fixation from the atmosphere and other biological parameters, especially protein content in the beans. Thus, the subsequent goal was to identify the source of the derived nitrogen (soil or atmospheric) in different parts of the plant, and to assess its accumulation and relocation in different growing stages (vegetative and reproductive) of soybean development. This was investigated in plants treated with different *Bradyrhizobium japonicum* strains and compared to organic fertilizer and endophytic applications. Measurements of nitrogen stable isotope ratios and nitrogen concentrations were conducted in different plant parts, including stems, shoots, and beans.

Therefore, the knowledge of how to improve the biological nitrogen fixation of rhizosphere bacteria, focusing on a single N_2_-fixing bacterium, can be important in further studies on potentially beneficial impacts of a bacterial consortium [15].

## 2. Results

### 2.1. Soybean Productivity at Flowering Stage R1

Data on biomass production of individual soybean plants and nodulation at the flowering stage of two soybean varieties, Laulema and Merlin, under different treatments revealed variety and treatment-dependent effects (Table 1). Statistical differences (one-way ANOVA with post hoc Tukey’s HSD test) were identified for all biological parameters under different treatments.

Biomass ranged from 97.81 g in Laulema control to 202.21 g on average in Laulema treated with *AGF78*, with inoculation resulting in a twofold increase. The weight proportion of one plant’s air-dried aboveground mass also increased to a similar extent, from 1.6 g on average in the control to 2.83 g on average in plants treated with *AGF78*; however, fertilization also caused the same increase and did not differ significantly from the inoculation treatments. This shows a significant effective fertilizer replacement by *Bradyrhizobium japonicum* inoculation.

Nodulation was only found in inoculated plants, but with different variability. Both varieties treated with *AGF78* inoculum exhibited the highest nodule numbers (up to 17 nodules per plant on average on Merlin, and up to 12 on Laulema) and the highest dry weight of nodules, accordingly. Only very early maturing Laulema treated with *RF10* showed statistically significant reductions in nodule and dry nodule weight per plant, indicating that it was less effective in nodulation. Meanwhile, *RF10* was effective, but to a lesser extent, for later-maturing Merlin.

Enhanced biological nitrogen fixation in inoculated plants was demonstrated by markedly lower δ^15^N values and higher nitrogen content in different parts of the plants (Table 2). Nitrogen stable isotope ratios varied significantly in shoots and roots during the early development stage of soybean plants, when the nodules were still forming, showing a direct response to the nitrogen isotope sources as well as nitrogen allocation between different parts of the plants. The exception was Laulema treated with *RF10*. This case had a tendency towards lower δ^15^N values, but it was still relatively high due to lower nodule number and weight. Additionally, the time required for N transfer to plant tissues showed the observable differences. Specifically, calculated %NDFA reached up to 77.8% on average in Laulema and 74.4% in Merlin treated with *AGF78*, resulting in notable contributions of fixed nitrogen (up to 37.4 kg/ha). In contrast, control plants, as well as those treated with *END2490* and fertilizer, showed no nodulation and relied entirely on soil nitrogen, as indicated by higher δ^15^N values and %NDFA values of zero. Treatment with *RF10* also positively influenced nodulation and nitrogen fixation, though to a slightly lower extent than *AGF78*.

Overall, application of *AGF78* inoculum significantly enhanced biomass accumulation, individual plant biomass, and nodulation of both Laulema and Merlin, compared to the control. *SEMIA507* and *RF10* were also effective; however, not all the improvements were statistically significant. These findings underscore the potential of microbial inoculants, particularly *AGF78*, to improve soybean growth performance and biological nitrogen fixation efficiency, with responses varying between varieties.

### 2.2. Soybean Yield Parameters

**Figure 1:**
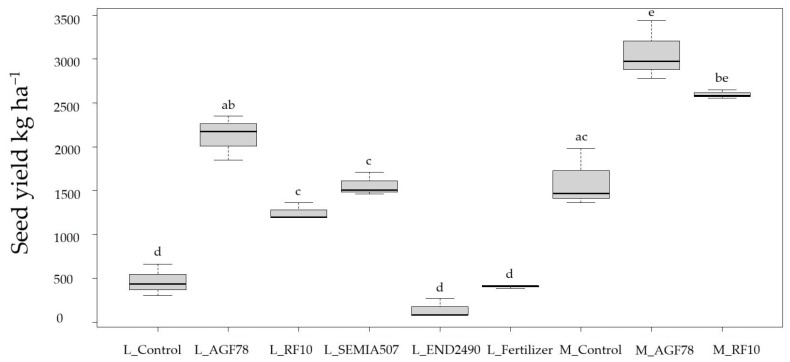
Soybean seed yield (kg/ha) in two varieties of soybean; the same letters show no statistically significant difference (*p* ≤ 0.05). Notes: L—Laulema, M—Merlin.

Based on the analysis of seed yield, grain weight, pod weight, and whole biomass (Table 3), notable differences were observed between the two varieties of soybean under various treatments. *Bradyrhizobium japonicum* strain *AGF78* consistently led to the highest enhancements in both varieties, particularly enhancing seed yield and grain weight, with later-maturing Merlin achieving the top bean yield of 3066.89 kg/ha on average (Figure 1). Merlin inoculated with *AGF78* showed the highest yield in overall cases. Similarly, inoculum *RF10* showed strong results, especially for Merlin.

**Figure 2:**
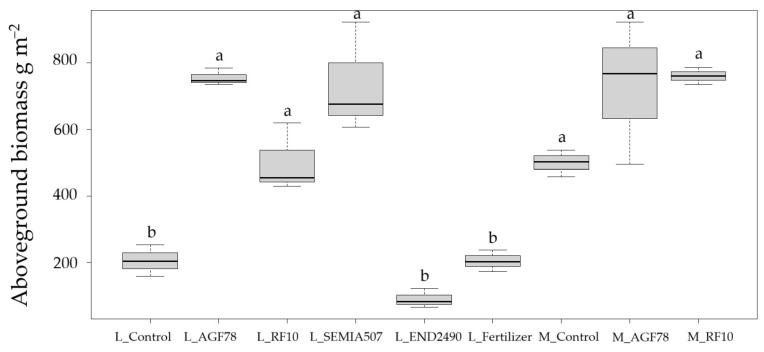
Aboveground biomass, g m^−2^ without roots in two varieties of soybean; the same letters show no statistically significant difference (*p* ≤ 0.05). Notes: L—Laulema, M—Merlin.

For Laulema, almost all the biological parameters improved significantly with any inoculum (except pod weight with *RF10*). In Merlin, seed yield enhancement was statistically significant; however, improvements with inoculums were observed to some extent in all cases. Aboveground biomass was significantly higher in inoculated treatments for Laulema, as well as all in treatments for Merlin (Figure 2). In contrast, the endophyte *END2490* performed poorly across all measured parameters, and no significant improvements were observed, indicating a limited or even negative impact. At the full maturity stage, R8, organic fertilizer did not yield any improvement and did not differ from the control. The control treatments showed lower productivity, particularly in Laulema, whose productivity was almost four times lower than with the *AGF78* treatment. The differences were statistically significant across all investigated parameters. *SEMIA507* was applied only to Laulema; however, it also yielded significant improvements.

These findings highlight a clear interaction between variety and treatment, demonstrating that the effectiveness of microbial or fertilization N inputs is variety-specific, and suggesting that inoculation is a promising tool for improving productivity in these crops.

### 2.3. Nitrogen Fixation and N Origin in Soybean Plant Parts

Table 4 and Table 5 present nitrogen stable isotope ratios, nitrogen percentages, as well as the calculated nitrogen derived from N_2_ fixation, as indicated by ^15^N natural abundance of plant samples (%NDFA), and Ndfa kg ha^−1^ in stems and beans of fully matured soybean plants. Nitrogen stable isotope ratio data showed that inoculated plants had lower δ^15^N values, indicating effective nitrogen fixation from the atmosphere, although statistical differences were not consistently significant.

Both control treatments had relatively high δ^15^N values compared with inoculated plants, showing high N uptake from mineral forms of the soil. The *END2490* and fertilizer treatments even exceeded these values, clearly showing stimulation of N uptake from the soil sources and manure itself. Also, δ^15^N values tended to be higher in beans compared with stems. In inoculated cases, δ^15^N values were significantly lower compared with the control, which were closer to atmospheric N-stable isotopic ratios, but the values did not vary a lot between the *Bradyrhizobium japonicum* strains and did not show statistically significant differences between them.

Generally, nitrogen content in beans was considerably higher and exceeded by up to 10 times compared with stems. In the case of Laulema, nitrogen content in beans was statistically significantly higher in inoculated plants; these tendencies persisted in Merlin, too. However, in stems, these differences were not observed.

Calculations of %NDFA show that inoculation with *AGF78* and SEMIA507 significantly enhanced nitrogen fixation in stems; however, *RF10* was not very effective in terms of BNF. Notably, the Merlin inoculated with *AGF78* resulted in the highest nitrogen fixation in beans, with 66.8%NDFA and 134.0 kg/ha of fixed nitrogen. Similarly, Laulema inoculated with *AGF78* resulted in 88.2%NDFA and 123.2 kg/ha of fixed nitrogen. Merlin inoculated with *RF10* also showed high NDFA values and substantial nitrogen fixation. Treatments with *END2490* and fertilizer did not promote nitrogen fixation.

Overall, the data demonstrate that effective inoculants, particularly *AGF78* in Laulema and Merlin and *RF10* in Merlin, can significantly increase biological nitrogen fixation in soybean, especially in the longer growing and later-maturing variety Merlin.

### 2.4. Quality Parameters of Beans

In terms of protein content, inoculation had a noticeable positive effect, particularly for Laulema (Table 6). The highest protein content in inoculated treatments was recorded for Laulema, which was treated with *RF10* (41.00 ± 0.51% on average), followed by treatment with SEMIA507 (40.05 ± 0.53% on average) and *AGF78* (38.10 ± 0.89%), indicating that selected inoculants significantly enhanced nitrogen assimilation and protein synthesis. Merlin also showed improvement, though to a lesser extent, with *RF10* and *AGF78* treatments reaching protein contents of 38.19 ± 0.19% and 37.40 ± 0.56%, respectively. Control treatments for both varieties had notably lower protein levels, confirming the clear benefit and necessity of inoculation.

The inverse relationship between protein and lipid content was expressed in most cases. Overall, treatments that increased protein levels tended to reduce lipid concentrations, suggesting that nitrogen metabolism may influence the allocation of resources between protein and oil synthesis in soybean seeds.

Meanwhile, 1000-seed weight also varied significantly across different treatments. The largest seed weight was observed for Laulema treated with fertilizer (226.02 ± 4.96 g on average), *AGF78* (223.38 ± 4.69 g on average), and *RF10* (214.13 ± 25.14 g on average), suggesting improved seed development in inoculated plants; however, not all the differences were statistically significant. While Merlin generally produced smaller seeds, the treatment with *AGF78* resulted in a particularly low seed weight (163.61 ± 2.33 g on average), although differences were not statistically significant; however, these trends indicate that not all inoculants were equally beneficial across varieties.

In summary, inoculation with selected bacterial strains had a positive impact on protein content and, in many cases, on seed weight, particularly in the Laulema. However, treatment effects varied and appeared to be both strain- and variety-specific. The observed trade-offs between protein and lipid accumulation highlight the complexity of metabolic regulation in soybean and underscore the need for careful inoculant selection to meet specific production goals.

## 3. Discussion

Inoculation plays a crucial role in improving nitrogen fixation and yield of legumes, though the benefits of inoculation vary with environment, soil conditions, and management practices [16,17]. Our study has proved its importance and demonstrated the scale of usefulness of symbioses between nodule-forming bacteria and soybean. For soybean, *Bradyrhizobium japonicum* inoculation is far more efficient in fields without a recent soybean history, where it ensures nodulation and significantly reduces the need for nitrogen fertilizers [10,14,18]. In Lithuania, most of the soils are free from soybean nitrogen-fixing bacteria; thus, to achieve BNF, additional inoculation with *Bradyrhizobium* sp. has to be applied.

Tracer methods are among the most precise approaches for monitoring inoculation effectiveness. However, it is easier to detect N changes using isotopic labeling techniques instead of natural abundance tests. In our study, natural abundance stable isotope differences were enough to monitor N sources and distinguish between inoculated and non-inoculated plants. The ^15^N natural abundance method, in turn, works best when (1) there are only two nitrogen sources for legume growth—soil nitrogen and atmospheric N_2_, (2) these sources have distinct ^15^N levels for accurate measurement, and (3) biological variability in ^15^N is minimal compared with the difference between the sources [19]. Meanwhile, more detailed studies confirm that soybeans are very efficient in BNF; therefore, it is easier to detect Ndfa while using various management practices [20,21].

In general, soybean cultivation is expanding in Central Europe due to the development of early-maturing cultivars and growing demand for plant-based protein produced without the use of genetically modified organisms [20]. Previously, it was not recommended to grow soybeans further than 54° North; however, recent studies in Lithuania, Scotland, or Denmark show that soybeans can be effectively grown above the northern boundary of their typical distribution [11,13,22]. However, these soils usually lack nodule-forming nitrogen-fixing bacteria; thus, additional inoculation is needed. Moreover, it is essential to look for effective strains that can improve soybean nitrogen fixation under the conditions prevailing in the region where the soybean crop will be grown, as strains isolated from cold environments can form nodules and bacteroides under low temperatures, whereas strains isolated from a warmer environment cannot [23]. In Scotland [11], inoculation doubled the grain yield compared with the non-inoculated crop when commercial inoculants containing elite *Bradyrhizobium japonicum* strains were used with a soybean 000 maturity group variety (ES Comandor). It also significantly increased plant biomass in plot trials. In our study, the experimental site was located above the northern boundary of soybean distribution, i.e., 55°24′ N, in a temperate climatic zone, and these conditions were appropriate for soybean cultivation and effective BNF. In the study site and year, aboveground biomass of soybean plants was almost double in inoculated plants compared with the control, and seed yield was up to four times higher than the control in the best affected cases. Inoculated soybeans increased nodulation, plant biomass, BNF, and yield components compared with non-inoculated soybeans.

Unfavorable climatic conditions for soybean cultivation are shortening of the active growth period, a delay of the date on which the soil warms up to 8 °C at a depth of 5 cm, occurrences of droughts, and late spring ground frosts [24]. Therefore, the role of climatic conditions for soybean cultivation is critical, and appropriate management practices have to be selected. An option for better crop management is integrated crop–livestock systems (ICLS), which combines crop cultivation with animal husbandry in a complementary manner, creating closed nutrient loops and enhancing system resilience. It has the potential to augment soybean production by modulating the microbial structure of soil [25].

Soybean productivity is influenced by complex interactions between genetic, climatic, and management factors. In the literature, the key determinants include cultivar selection, soil fertility, climate conditions, and crop management practices, such as planting density, intercropping, irrigation, or pest control [26]. According to Toleikienė et al. [6], the yield quality of soybean grown in Lithuania was significantly affected by inoculation, row spacing, sowing time, and the interactions among them. A study in Germany [9] showed significantly higher nodule numbers and nodule dry weights following selected inoculation in well-watered soil, but only minor differences under drought conditions. Inoculation of the soybean cultivar Merlin with the selected strain enhanced nodulation but did not correspond to an increased grain yield under field conditions. A study in Denmark [13] proposed additional inoculation at the V_3_ growth stage, which is defined by having three fully developed, unrolled trifoliate leaves, as a promising treatment to improve nodulation in soybean plants.

A study in Poland [20] showed that nodulation varied significantly across years, with the highest values recorded under favorable early-season moisture and reduced values during drought. Other studies [21] showed that seed inoculation with nitrogen-fixing bacteria improved the grain yield, biological yield, oil content, and protein content of soybean, as compared with non-inoculated controls. To optimize conditions for soybean cultivation, different strategies were proposed, like combining inoculation with nitrogen fertilizers [27]. The experimental treatments included native Bradyrhizobia, commercial *Bradyrhizobium japonicum*, and a mixture of both. The results demonstrated significant improvement in soybean nodule dry weight, shoot dry weight, and seed dry weight following bradyrhizobia inoculation. Remarkably, organic farming significantly outperformed conventional systems in nodulation, which is a very promising finding as organic farming is gaining increasing attention [28]. However, studies comparing the effects of different strains of *Bradyrhizobium japonicum* under Northern European conditions are historically relevant but rare [29], and existing studies were frequently conducted in other continents more than a decade ago [30]. Therefore, our study provides essential information by comparing new strains of *Bradyrhizobium japonicum* and soybean varieties, recognizing the importance of Northern European conditions.

Significant advancements in soybean development have been achieved through genetic breeding programs, which have increased yield by selecting genotypes adapted to the edaphoclimatic conditions of each region [31]. In the genotypes evaluated, oil content exhibited a negative correlation with protein content, whereas yield demonstrated a positive correlation with plant size [32]. In our study, we studied two varieties of soybean and different commercially available products, containing nitrogen-fixing *Bradyrhizobium japonicum* bacteria, and found their impact on biological parameters to be a very promising practice to enhance BNF. Compared with the other neighboring countries, our yields are similar. For example, a study in Poland [20] shows that the highest seed yield was recorded in 2019 (3.69 ± 0.04 t·ha^−1^), while the lowest was observed in 2017 (2.26 ± 0.07 t·ha^−1^). Seed yield increased progressively with higher nitrogen rates, from 2.77 ± 0.12 t·ha^−1^ at 0 kg N·ha^−1^ to 2.97 ± 0.11 t·ha^−1^ at 60 kg N·ha^−1^. In our case, the seed yields around 3 t·ha^−1^ on average in case of Merlin; however, the results can be variable depending on the variety selection.

Dinitrogen (N_2_) is exceptionally stable due to its strong triple bond, which makes industrial fertilizer synthesis energy-intensive, economically costly, and environmentally burdensome. The Haber–Bosch process is currently one of the largest global energy consumers and greenhouse gas emitters, responsible for 1.2% of the global anthropogenic CO_2_ emissions, leading researchers to recommend alternative production methods [33]. In contrast, biological nitrogen fixation by the nitrogenase enzyme complex reduces N_2_ to ammonia under ambient conditions, providing a more energy-efficient natural pathway [34]. Synthetic nitrogen fertilizers are not always applied when growing soybeans, but they can affect biological parameters like yield and seed composition, and the outcomes of N fertilization varied among studies [35]. In our study, we used organic fertilizer, which positively affected biomass, but not seed yield; it also had no significant effect on protein concentration in beans; however, the 1000-seed mass was higher. Other studies [35] found wide variability in the response of soybean seed protein concentration to nitrogen fertilization, with the soybean physiology, seed yield, and composition being a function of genetics, environment, and management practices. Nitrogen application showed significant interactions with the environment for seed protein concentration, seed oil concentration, yield, and the percentage of nitrogen derived from the atmosphere in leaves during the seed fill period. The effect of nitrogen on protein concentration varied across environments, with an increase observed in one environment and a decrease in another. Therefore, environment-specific nitrogen management is the most effective strategy for improving seed protein in this region characterized by diverse environmental conditions [36]. Overall, long-term co-application of rhizobium and fertilizer not only increased soybean yield, but also altered soil bacterial community structure through niche reconstruction and microbial interaction. Rhizobium inoculation plays a key role in reducing nitrogen fertilizer application and promoting sustainable agriculture practices [37].

In general, inoculation is a relatively low-cost, environmentally friendly strategy to support legume productivity, with the greatest gains in organic agriculture, under nutrient limitations, as well as first-time cultivation. However, further studies are necessary to test the survival of nitrogen-fixing bacteria and the effectiveness of symbiosis with different varieties of plants.

## 4. Materials and Methods

### 4.1. Study Area and Environmental Conditions

The field experiment was carried out in the Lithuanian Research Centre for Agriculture and Forestry (LAMMC) in Akademija (55°24′ N, 23°51′ E), Kėdainiai district. This region is situated beyond the soybean growing northern limit, at 55°24′ N. Lithuania falls within a cool temperate climate zone, where the average annual temperature is 6.5 °C, and the growing season ranges between 169 and 202 days. Field trials were performed in the 2022 cropping season in an organically managed site. The selected fields have been managed organically since 2003, with no additional irrigation, pesticides, or chemical inputs. It is dedicated exclusively to crop production, with nitrogen supplied through a diverse mix of grain and forage legumes, plant-based fertilizers, and microbial products.

The soil of the experimental site was a loamy Endocalcaric Epigleyic Cambisol (Drainic, Loamic) CM-can.glp dr.lo. The arable layer (0–25 cm) was described by a pH of 7.5, 77 mg·kg^−1^ available P_2_O_5_, 2.4% humus content, and 138 mg·kg^−1^ potassium.

**Figure 3:**
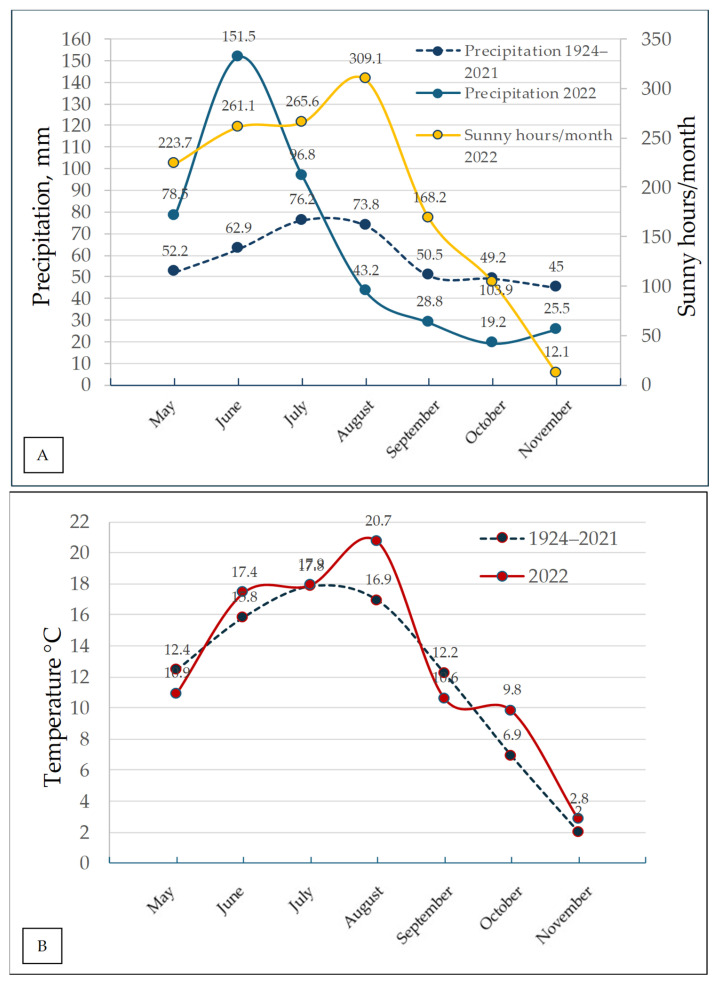
(**A**) Precipitation in 2022 compared with the 1924–2021 average and monthly sunshine hours in 2022; (**B**) temperature during the soybean cultivation period in 2022 compared with the long-term average (1924–2021) in Akademija.

Weather data were collected at the stationary meteorological station, located in Akademija, using the temperature and rainfall sensors. Precipitation, temperature, and sunny hours data are presented in Figure 3.

Spring in 2022 exhibited variable weather patterns, characterized by a colder-than-average May. Sunny and warm conditions predominated during the season, except for May. May recorded the lowest temperatures. The mean air temperature for the spring months was 0.3 °C above the long-term average (5.9 °C). Total precipitation for the season amounted to 135 mm, representing 115% of the long-term average (1924–2021) (116.9 mm).

The summer of 2022 was windy, warm, and rainy, except for August. The average air temperature during the summer months was 1.8 °C higher than the long-term average (16.8 °C). Total precipitation amounted to 297.5 mm, which was 140% of the long-term average (212.1 mm). A rainy period occurred during the first and second ten-day intervals of June, while August was dry. Overall, the weather was favorable for harvesting, which began two weeks earlier than usual.

Autumn was relatively warm, dry (except for September), and windy. The average air temperature was 0.7 °C higher than the long-term average (7.0 °C). Precipitation was 73.5 mm, which was 51% of the long-term average (144.7 mm).

### 4.2. Experimental Design and Treatments

The field experiment was conducted in a randomized complete block design with three replications to ensure statistical reliability and minimize the effects of variability within the field. Each plot was 1.5 m wide and 5.0 m long, with a total area of 7.5 m^2^.

Two soybean varieties, showing good yields in Lithuania in the previous years, were selected for further analysis, according to their maturity group. ‘Laulema’ belongs to the very-early variety (0000-maturity group), while ‘Merlin’ is classified as an early (000-maturity group) variety. Prior to sowing, the soybean seeds were inoculated (product containing nitrogen-fixing bacteria was applied to the seeds and dried in room temperature) with three commercially available products containing *Bradyrhizobium japonicum* strains: HiStick^®^Soy (BASF, Ludwigshafen, Germany), containing 4 × 10^9^ viable cells/g of strain SEMIA507 (hereafter, SEMIA507), BACTOLiVE^®^ (RHIZO-MIC UG, St. Johann, Germany) containing 1 × 10^9^ colony-forming units of strain AGF78 per gram of peat material (CFU/g) (hereafter, as AGF78), and RhizoFix^®^ RF-10 (Feldsaaten Freudenberger GmbH & Co. KGcontaining, Krefeld, Germany) 1 × 10^9^ colony-forming units of strain RF10 in a ml of liquid inoculant (CFU/mL) (hereafter, as RF10). Additionally, one treatment was inoculated with a commercial endophyte (hereafter, as END2490). For control, one treatment was not inoculated and received no fertilization (control), and one treatment was not inoculated but fertilized with organic manure (granulated chicken manure). The rate of granulated chicken manure was calculated to deliver 45 kg/ha N.

Soybean seeds were sown on 15 May 2022, using 12.5 cm interrow spacing and a sowing rate of 80 viable seeds per m^−2^. The soil was plowed in the autumn and harrowed before sowing. Spring wheat was the pre-crop. The soybean plant samples were collected twice during vegetation: at the flowering stage R1 (21 July 2022), and at the full maturity stage R8 (9 and 28 September). Twenty full plants with undisturbed roots and complete nodule systems were dug up during the first sampling. During the second sampling, all the plants from 4 × 0.25 m^−2^ subplots were harvested. Shoots, roots, and beans of each plant were counted, measured, weighed, and dried at 60 °C for 36 h, then ground in a mill to a fine powder and stored in a dry place prior to analyses. Plots were harvested for grain yield and yield quality data on 9 September for Laulema and on 28 September for Merlin. Bean samples were analyzed for 1000-kernel weight and protein content using a grain analyzer, Infratec 1241 (Foss, Hilleroed, Denmark).

### 4.3. Natural Abundance Stable Isotope Technique

Homogenized shoot, stem, and bean samples were weighed in tin capsules for stable isotope analysis. Nitrogen stable isotope ratio measurements of the prepared samples were performed using the elemental analyzer Thermo Fisher Scientific, FlashEA 1112 (Delft, The Netherlands), connected to the Thermo Fisher Scientific Finnigan Delta Advantage (Bremen, Germany) isotope ratio mass spectrometer (IRMS). All stable isotope ratio measurements were expressed relative to a standard (atmospheric air N_2_) using delta (δ) notation, reported in parts per thousand (‰):

δ^15^N = (R_sample_/R_standard_ − 1) × 10^3^

where R = ^15^N/^14^N in the sample or in the standard. During the analysis, samples were interspersed with several replicates of laboratory reference materials, calibrated against international reference materials, including IAEA-N-1, IAEA-600, provided by the International Atomic Energy Agency (IAEA). The long-term standard deviation is <0.2‰; however, a subset of the soybean samples (~10%) was measured in duplicate to assess sample homogeneity, and the resulting values were pooled.

The percentage of N derived from the atmosphere (%Ndfa) was calculated according to Unkovich et al. [38]; this method takes advantage of the naturally higher ^15^N concentration of the N available in the soil compared with atmospheric N_2_.

### 4.4. Statistical Analysis

Isotopic values are reported as means ± standard deviations (SD). Statistical analysis of the impact of inoculation on soybean yield and other biological parameters was performed using one-way ANOVA with Tukey’s post hoc test for mean comparisons across all treatments in R Commander (version 4.2.2). Differences were considered statistically significant at *p* < 0.05.

## 5. Conclusions

Overall, soybean inoculation with selected bacterial strains in most treatments had a positive impact on various biological parameters of the plants, like biomass, seed yield, and weight, as well as nitrogen and protein content and weight of the seeds. However, treatment effects varied and appeared to be both strain- and variety-specific. Inoculation improved biological parameters in Laulema to a greater extent compared with Merlin. Moreover, the study showed that inoculation can have different effects on biological parameters; thus, before inoculation, the target biological parameters have to be considered, and an appropriate strategy has to be selected.

Biological N fixation was only present when inoculants were applied; therefore, fertilization and endophytic treatments relied solely on soil or applied nitrogen sources. Both varieties, Laulema and Merlin, inoculated with AGF78 showed significantly higher %NDFA, especially in beans (88.2% and 66.8%, respectively); however, other inoculation treatments promoted N fixation, but less effectively than AGF78.

The highest efficiency was achieved by Merlin, showing the highest quantity of nitrogen fixed per hectare (134.0 kg/ha). Further research is needed to determine which additional varieties and inoculants may be effective under changing and less favorable environmental conditions.

## Figures and Tables

**Table 1:** Biological parameters in inoculated and non-inoculated treatments of soybean varieties, Laulema and Merlin, at the flowering stage.

Variety/Treatment	Biomass—Weight of Air-Dried Aboveground Mass, g m^2^	Weight of 1 Plant’s Air-Dried Aboveground Mass, g	Nodule Number per Plant	Weight of Dry Nodules per 1 Plant, g
Laulema/Control	97.81 ± 1.60 ^a^	1.6 ± 0.03 ^a^	0	0
Laulema + *AGF78*	202.21 ± 11.7 ^b^	2.83 ± 0.16 ^b^	12 ± 3 ^ab^	0.1100 ± 0.0020 ^a^
Laulema + *SEMIA507*	149.03 ± 41.46 ^ab^	2.00 ± 0.56 ^ab^	10 ± 4 ^ab^	0.0613 ± 0.0231 ^b^
Laulema *+ RF10*	120.80 ± 18.09 ^a^	2.04 ± 0.30 ^ab^	8 ± 2 ^a^	0.0587 ± 0.0150 ^b^
Laulema + *END2490*	115.42 ± 44.34 ^a^	1.83 ± 0.70 ^ab^	0	0
Laulema + Fertilizer	152.71 ± 22.83 ^ab^	2.83 ± 0.42 ^b^	0	0
Merlin/Control	144.87 ± 20.99 ^ab^	2.22 ± 0.32 ^ab^	0	0
Merlin + *AGF78*	150.21 ± 22.92 ^ab^	2.42 ± 0.37 ^ab^	17 ± 6 ^b^	0.1053 ± 0.0390 ^ab^
Merlin + *RF10*	150.29 ± 23.39 ^ab^	2.23 ± 0.35 ^ab^	14 ± 1 ^ab^	0.0920 ± 0.0100 ^ab^

Means followed by the same letters in the same column do not significantly differ from one another (*p* ≤ 0.05).

**Table 2:** Nitrogen stable isotope ratios, nitrogen content, and NDFA of soybean.

Variety/Treatment	δ^15^N, ‰ Shoots	N, % Shoots	%NDFA	Ndfa, kg ha^−1^
Laulema/Control	5.65 ± 2.23 ^ab^	1.43 ± 0.08 ^a^	0	0
Laulema + *AGF78*	1.25 ± 0.23 ^a^	2.46 ± 0.18 ^bcd^	77.8 ± 4.1 ^a^	37.0 ± 5.0
Laulema + SEMIA507	1.30 ± 0.25 ^a^	2.88 ± 0.14 ^d^	76.9 ± 4.4 ^a^	37.4 ± 11.8
Laulema *+ RF10*	5.02 ± 1.43 ^ab^	1.99 ± 0.18 ^abc^	29.0 ^b^	9
Laulema + *END2490*	6.56 ± 1.05 ^b^	1.69 ± 0.15 ^a^	0	0
Laulema + Fertilizer	6.48 ± 0.03 ^b^	1.70 ± 0.31 ^ac^	0	0
Merlin/Control	6.44 ± 2.40 ^b^	1.94 ± 0.32 ^ac^	0	0
Merlin + *AGF78*	1.65 ± 0.01 ^ab^	2.54 ± 0.08 ^bd^	74.4 ± 0.2 ^a^	26.8 ± 6.3
Merlin + *RF10*	3.04 ± 0.79 ^ab^	2.34 ± 0.18 ^bd^	52.9 ± 12.3 ^ab^	20.6 ± 0.0

Means followed by the same letters in the same column do not differ significantly from one another (*p* ≤ 0.05).

**Table 3:** Seed yield, bean weight, pod weight, and total plant biomass of inoculated and non-inoculated soybean varieties, Laulema and Merlin.

Variety/Treatment	Seed Yield of 13% Moisture, kg ha^−1^	Bean Weight, g m^−2^	Pod Weight (Grain-Free), g m^−2^	Aboveground Biomass, g m^−2^
Laulema/Control	465.59 ± 182.16 ^d^	101.41 ± 25.64 ^b^	61.53 ± 14.26 ^bd^	205.71 ± 46.90 ^b^
Laulema + *AGF78*	2124.87 ± 256.02 ^ab^	405.77 ± 16.08 ^a^	207.85 ± 4.52 ^a^	755.69 ± 25.76 ^a^
Laulema + *SEMIA507*	1560.86 ± 133.46 ^ac^	390.00 ± 74.35 ^a^	208.08 ± 39.12 ^a^	735.75 ± 166.27 ^a^
Laulema + *RF10*	1250.44 ± 97.99 ^c^	267.40 ± 62.77 ^a^	128.14 ± 36.37 ^bc^	501.45 ± 103.67 ^a^
Laulema + *END2490*	143.92 ± 109.14 ^d^	44.71 ± 13.05 ^b^	25.61 ± 8.52 ^d^	90.08 ± 29.07 ^b^
Laulema + Fertilizer	404.83 ± 15.95 ^d^	88.19 ± 11.58 ^b^	70.25 ± 9.35 ^bd^	204.83 ± 32.20 ^b^
Merlin/Control	1603.91 ± 333.20 ^ac^	258.51 ± 20.43 ^a^	127.61 ± 12.99 ^bc^	499.67 ± 40.03 ^a^
Merlin + *AGF78*	3066.89 ± 338.40 ^e^	402.72 ± 124.37 ^a^	190.85 ± 48.78 ^ac^	729.48 ± 216.15 ^a^
Merlin + *RF10*	2596.53 ± 49.74 ^be^	413.44 ± 9.32 ^a^	185.53 ± 4.41 ^ac^	761.27 ± 25.50 ^a^

Means followed by the same letters in the same column do not differ significantly from one another (*p* ≤ 0.05).

**Table 4:** Nitrogen stable isotope ratios, nitrogen contents, %NDFA, and Ndfa kg ha^−1^ in stems of two varieties of soybean at the full maturity stage.

Variety/Treatment	δ^15^N, ‰ Stems	N, % Stems	%NDFA Stems	Ndfa, kg ha^−1^ Stems
Laulema/Control	1.9 ± 0.8 ^abc^	0.64 ± 0.10 ^ab^	0	0
Laulema + *AGF78*	1.0 ± 0.7 ^a^	0.64 ± 0.03 ^a^	45.6 ± 37.8 ^a^	4.0 ± 3.4 ^a^
Laulema + SEMIA507	0.7± 0.6 ^a^	0.63 ± 0.03 ^a^	63.8 ± 31.1 ^a^	5.6 ± 2.4 ^a^
Laulema + *RF10*	2.0 ± 0.3 ^abc^	0.92 ± 0.17 ^ab^	0	0
Laulema + *END2490*	3.0 ± 2.0 ^bc^	0.66 ± 0.14 ^a^	0	0
Laulema + Fertilizer	3.5 ± 1.1 ^b^	0.67 ± 0.14 ^a^	0	0
Merlin/Control	1.7 ± 0.8 ^abc^	1.12 ± 0.36 ^ab^	0	0
Merlin + *AGF78*	0.3 ± 0.5 ^ac^	0.85 ± 0.13 ^ab^	84.8 ± 30.4 ^a^	9.5 ± 4.9 ^a^
Merlin + *RF10*	1.2 ± 0.4 ^ac^	1.31 ± 0.25 ^b^	31.7 ± 23.2 ^a^	6.9 ± 4.7 ^a^

Means followed by the same letters in the same column do not differ significantly from one another (*p* ≤ 0.05).

**Table 5:** Nitrogen stable isotope ratios, nitrogen contents, %NDFA, and Ndfa kg/ha in beans of two varieties of soybean at the full maturity stage.

Variety/Treatment	δ^15^N, ‰ Beans	N, % Beans	%NDFA Beans	Ndfa, kg ha^−1^ Beans
Laulema/Control	2.23 ± 0.31 ^bc^	5.26 ± 0.21 ^c^	0	0
Laulema + *AGF78*	0.26 ± 0.18 ^ac^	6.18 ± 0.13 ^ab^	88.2 ± 8.1 ^a^	123.2 ± 7.0 ^b^
Laulema + SEMIA507	0.46 ± 0.29 ^a^	6.83 ± 0.23 ^ab^	79.2 ± 13.2 ^a^	85.9 ± 16.8 ^ab^
Laulema + *RF10*	0.55 ± 0.16 ^ac^	7.04 ± 0.16 ^a^	75.4 ± 7.0 ^a^	39.6 ± 36.4 ^a^
Laulema + *END2490*	3.19 ^bd^	4.95 ^c^	0	0
Laulema + Fertilizer	4.04 ± 0.64 ^d^	5.22 ± 0.12 ^c^	0	0
Merlin/Control	2.28 ± 1.18 ^b^	5.73 ± 0.45 ^cd^	0	0
Merlin + *AGF78*	0.76 ± 0.43 ^ac^	6.43 ± 0.25 ^bd^	66.8 ± 18.8 ^a^	134.0 ± 30.2 ^b^
Merlin + *RF10*	0.66 ± 0.13 ^ac^	6.91 ± 0.21 ^ab^	71.0 ± 5.6 ^a^	126.9 ± 11.9 ^b^

Means followed by the same letters in the same column do not differ significantly from one another (*p* ≤ 0.05).

**Table 6:** Proteins, lipids, and 1000-seed weight data in bean samples of Laulema and Merlin.

Variety/Treatment	Proteins, %	Lipids, %	1000 Seed Weight, g
Laulema/Control	33.06 ± 1.56 ^de^	19.45 ± 0.55 ^df^	190.48 ± 12.07 ^bcd^
Laulema + *AGF78*	38.10 ± 0.89 ^ab^	17.78 ± 0.53 ^a^	223.38 ± 4.69 ^a^
Laulema + SEMIA507	40.05 ± 0.53 ^ac^	16.50 ± 0.27 ^b^	204.37 ± 2.98 ^abc^
Laulema + *RF10*	41.00 ± 0.51 ^c^	16.38 ± 0.26 ^b^	214.13 ± 25.14 ^ab^
Laulema + *END2490*	32.23 ^de^	20.15 ^cd^	201.88 ± 12.91 ^abc^
Laulema + Fertilizer	30.88 ± 0.99 ^d^	20.87 ± 0.66 ^c^	226.02 ± 4.96 ^a^
Merlin/Control	33.91 ± 1.33 ^e^	18.88 ± 0.07 ^ef^	184.68 ± 3.51 ^bcd^
Merlin + *AGF78*	37.40 ± 0.56 ^b^	18.13 ± 0.028 ^ae^	163.61 ± 2.33 ^d^
Merlin + *RF10*	38.19 ± 0.0.19 ^ab^	17.71 ± 0.04 ^a^	181.08 ± 4.39 ^cd^

Means followed by the same letters in the same column do not differ significantly from one another (*p* ≤ 0.05).

## Data Availability

The original contributions presented in this study are included in the article. Further inquiries can be directed to the corresponding author.
